# Fluoride Exposure and ADHD: A Systematic Review of Epidemiological Studies

**DOI:** 10.3390/medicina59040797

**Published:** 2023-04-19

**Authors:** Gianluca Fiore, Federica Veneri, Rosaria Di Lorenzo, Luigi Generali, Marco Vinceti, Tommaso Filippini

**Affiliations:** 1 Environmental, Genetic and Nutritional Epidemiology Research Center (CREAGEN), Section of Public Health, Department of Biomedical, Metabolic and Neural Sciences, University of Modena and Reggio Emilia, 41125 Modena, Italy; 2 Unit of Dentistry & Oral-Maxillo-Facial Surgery, Department of Surgery, Medicine, Dentistry and Morphological Sciences with Transplant Surgery, Oncology and Regenerative Medicine Relevance (CHIMOMO), University of Modena and Reggio Emilia, 41124 Modena, Italy; 3 PhD Program in Clinical and Experimental Medicine, Department of Biomedical, Metabolic and Neural Sciences-University of Modena and Reggio Emilia, 41125 Modena, Italy; 4 Service of Psychiatric Diagnosis and Care (SPDC), Department of Mental Health and Drug Abuse, AUSL Modena, 41124 Modena, Italy; 5 Department of Epidemiology, Boston University School of Public Health, Boston, MA 02118, USA; 6 School of Public Health, University of California Berkeley, Berkeley, CA 94704, USA

**Keywords:** ADHD, fluoride, attention deficit hyperactivity disorder, neurobehavioral, systematic review, epidemiology

## Abstract

*Background and objectives*: Attention deficit hyperactivity disorder (ADHD) is a childhood-onset neurodevelopmental disorder characterized by two dimensions: inattentiveness and hyperactivity/impulsivity. ADHD may be the result of complex interactions between genetic, biological and environmental factors possibly including fluoride exposure. *Materials and methods*: A literature search was performed on 31 March 2023 in the following databases: PubMed, Embase and Web of Science. We defined the following inclusion criteria according to the PECOS statement: a healthy child and adolescent population (P), fluoride exposure of any type (E), comparison with low or null exposure (C), ADHD spectrum disorder (O), and ecological, cross-sectional, case–control and cohort studies (S). *Results*: We found eight eligible records corresponding to seven different studies investigating the effect of fluoride exposure on children and adolescents. One study had a cohort design and one a case–control one, while five were cross-sectional. Only three studies applied validated questionnaires for the purpose of ADHD diagnosis. As regards exposure assessment, levels of fluoride in urine and tap water were, respectively used in three and two studies, while two used both. Three studies reported a positive association with ADHD risk, all assessing exposure through fluoride levels. By using urinary fluoride, conversely, a positive correlation with inattention, internalizing symptoms, cognitive and psychosomatic problems was found in three studies, but no relation was found in the other one. *Conclusions*: The present review suggests that early exposure to fluoride may have neurotoxic effects on neurodevelopment affecting behavioral, cognitive and psychosomatic symptoms related to ADHD diagnosis. However, due to the heterogeneity of the studies included, current evidence does not allow to conclusively confirm that fluoride exposure is specifically linked to ADHD development.

## 1. Introduction

Attention deficit hyperactivity disorder (ADHD) is a childhood-onset neurodevelopmental disorder characterized by two dimensions: inattentiveness and hyperactivity/impulsivity [1]. The definition “Attention deficit disorder” was introduced in 1980 in the Diagnostic and Statistical Manual of Mental Disorders, Third Edition (DSM-III) [2], but symptoms attributable to ADHD have been described in the scientific literature since 1775 [3,4]. Nowadays, ADHD is defined as a neurodevelopmental disorder: it was revised on various occasions over the years, most recently in the Diagnostic and Statistical Manual of Mental Disorders, Fifth Edition (DSM-5) in 2013 [5]. ADHD is usually diagnosed in early childhood and adolescence, but it can persist as a lifelong, chronic disorder. Studies published after 2011 reported an increased prevalence of persistent ADHD in adulthood: 55% of individuals continue to have enduring and impairing symptoms in adult life [6,7].

Among children and adolescents, ADHD is one of the most common psychiatric disorders, with an estimated prevalence of 3.4% worldwide [8,9]. National Institute for Health and Care Excellence (NICE) guidelines suggest an increased prevalence of ADHD in particular conditions and/or comorbidities: people born preterm, looked-after children (those who have been in the care of local authorities for more than 24 h), children and adolescents diagnosed with oppositional defiant disorder, with conduct or mood disorders, with a close family member diagnosed with ADHD, epilepsy, neurodevelopmental disorders (autism spectrum disorder, tic disorders, learning disability), a history of substance misuse, acquired brain injury, and known to the Youth Justice System [10].

ADHD is the result of complex interactions between genetic, biological and environmental factors. Although the precise etiology of ADHD is still unknown, it has been suggested to be an inherited disorder, with heritability ranging between 70% and 80% [11,12]. Neurobiological theories mainly regard prefrontal cortex (PFC) impairment in ADHD: the reduction of the volume and activity of PFC is implicated in ADHD-related impulsivity and inattention, while a delayed maturation of PFC is linked to symptom persistence [13]. ADHD symptoms are linked to abnormalities in dopamine and norepinephrine signal pathways, which constitute the main therapeutic target of psychopharmacological drugs [14]. Alteration of serotonergic and cholinergic systems may also affect ADHD physiopathology [15]. Acetylcholine is a major neurotransmitter involved in learning, memory, and intelligence [16]. Alterations in nicotinic acetylcholine receptor (nAChR) function may play a part in the symptomatology of ADHD, and the stimulation of these receptors may relieve from ADHD symptoms [17]. Nicotinic cholinergic neurotransmission is involved in attention and executive function processes. Furthermore, nicotine has shown procognitive effects in a number of animal studies, while treatment using novel nicotinic agonists showed evidence of symptom improvement in adult ADHD [18].

Environmental factors might also play a role in the manifestation of ADHD as suggested by the increased prevalence of the disease over the last decades [19], possibly due to the diffusion of industrial chemicals and air pollutants impairing brain development [20,21]. Other etiological factors have been linked to ADHD, such as tobacco/alcohol use during pregnancy, low birth weight and early psychosocial adversities such as severe marital discord, paternal criminality or maternal mental disorder [22]. Moreover, heavy metal and chemical exposures (lead, organochlorines, air pollution, mercury) have been associated with ADHD development. Nevertheless, the actual involvement of these environmental contaminants is still controversial [23,24,25,26].

Among other compounds, the trace element fluoride has been investigated for its possible neurotoxic effects [27,28]. Inorganic fluoride can be found in nature in different amounts in food, plants and drinking water. Moreover, fluoride has long been intentionally used for dental caries prevention and management through community-based strategies (e.g., water, milk, salt fluoridation), drops or tablet supplementation, or topical applications (e.g., toothpastes, mouth rinses, gels, and varnishes) [29]. Fluoride exposure mostly comes from fluoridated water and foods and beverages prepared with fluoridated water, although the accidental ingestion of fluoride-containing dental products can account for a small part of the intake [30]. Early exposure to fluoride has been associated with impaired cognitive neurodevelopment with low intelligence quotient (IQ). This association has been reported starting at rather low levels of fluoride exposure such as 1 mg/L in water, which can also be detectable in urine at corresponding levels [31,32]. As matter of fact, also the National Toxicology Program (NTP) aimed to explore the relation between fluoride exposure and neurodevelopmental and cognitive health effects in its recent systematic review. NTP concludes that fluoride might be considered as a cognitive neurodevelopmental hazard to humans. This conclusion is based on the consistent pattern of findings in human studies across several different populations, showing that higher fluoride exposure is associated with decreased IQ or other cognitive impairments in children [33].

This potentially harmful effect can be explained at two levels. First of all, the ability of fluoride to cross the less efficient blood–brain barrier in prenatal and early life. Secondly, the ability to concentrate in the brain areas responsible for memory and learning abilities, affecting the metabolism and physiology of neuronal and glial cells through oxidative stress [34,35]. Fluoride overexposure can suppress thyroid function regardless of a possible concurrent iodine deficiency, leading to the development of hypothyroidism [36,37]. Thyroid hormones play an essential role in the metabolic homeostasis of the nervous system, especially during the early phases of neurodevelopment in both fetuses and newborn children [38]. Hippocampal synaptic structures specifically involved in the development of verbal and visual-spatial skills are also highly sensitive to thyroid hormone levels as well as direct fluoride exposure [39,40]. Similarly, some evidence of fluoride adverse effects on behavioral alterations and neurodevelopmental disorders has been reported [41,42]. The aim of this study was to review epidemiological evidence on the association between fluoride exposure and ADHD risk.

## 2. Materials and Methods

This systematic review was registered in PROSPERO (no. CRD42022321899) and was conducted in accordance with the recommendations of the PRISMA (The Preferred Reporting Items for Systematic reviews and Meta-Analyses) statement. The research question following the PECOS (Population, Exposure, Comparison, Outcome, Study design) statement was “What is the association between early or prenatal fluoride exposure on ADHD risk in non-adult populations as assessed in non-experimental studies?”. As a consequence, inclusion criteria were as follows: a healthy child and adolescent population (P), fluoride exposure of any type, e.g., water, food, biomarker (E), comparison to null or low exposure (i.e., the lowest level of fluoride concentration reported in each study included) (C), ADHD spectrum disorder as an outcome (O), and ecological, cross-sectional, case–control and cohort studies (S).

We conducted a literature search in the following databases on March 31, 2023: PubMed, Embase and Web of Science. We used MeSH terms and keywords related to “ADHD” and “fluoride”. Full search strategies are reported in Table 1.

No limits were set to data or language. Reference lists from the retrieved articles have also been searched. Title and abstracts of the remaining records were screened against inclusion criteria by one author (GF), involving another (TF) in case of uncertainty, while those found to be irrelevant were discarded. Full texts of the remaining records were independently obtained and reviewed by two authors (GF and TF). In case of disagreement between the two reviewers, this was resolved by discussion until consensus was reached.

## 3. Results

**Figure 1:**
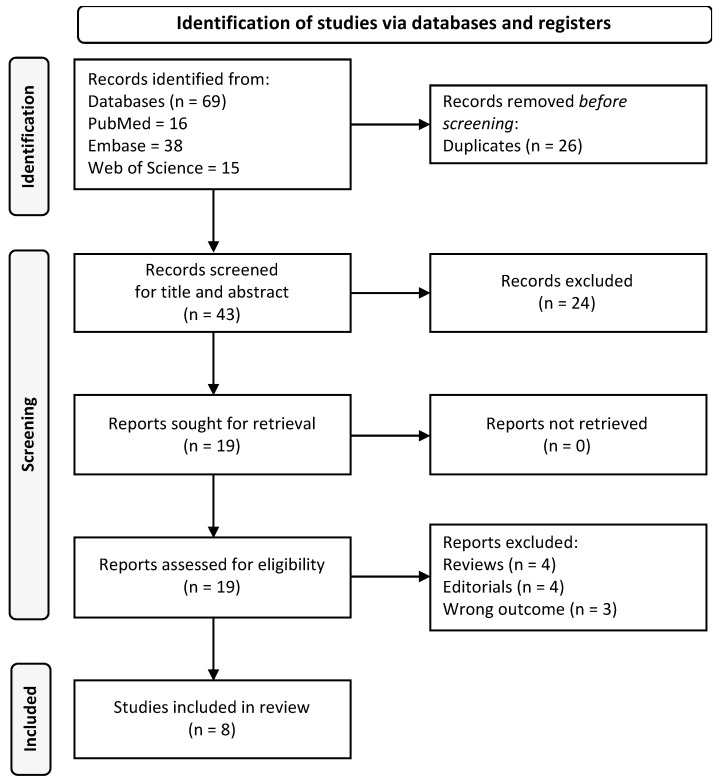
PRISMA flow-chart of the inclusion process.

A total of 43 records were retrieved after duplicate removal. We excluded 24 records based on title and abstract screening and further excluded 11 records due to full-text evaluation (4 reviews, 3 editorials and 4 articles with different outcomes). Finally, eight eligible papers were included in this review. Figure 1 shows the flow chart of the selection process.

These eight papers referred to seven studies. One had a cohort design and another a case–control one, while five studies were cross-sectional. Two studies were conducted in the USA, two in Canada, one in Mexico, one in China and one in India. Table 2 shows the main features of the studies included in the review.

Each study used different methods to assess fluoride exposure and diagnose ADHD. This also leads to remarkable heterogeneity in the results presented. In particular, Bashash et al., 2018 [43], Riddell et al., 2019 [42], Adkins et al., 2022 [46], Wang et al., 2022 [47], and Barberio et al., 2017 [48] collected information on urinary fluoride levels, whereas fluoride water levels are taken into account in the studies by Malin and Till 2015 [44] and Kairkar et al., 2021 [49]. In particular, the ecological design in Malin and Till 2015 was instrumental in collecting data from the Center for Disease and Control and Prevention (CDC) regarding the number of people receiving fluoridated water from public supplies in America’s 50 States and the Columbia District in 1992, 2000, 2002, 2004, 2006 and 2008. Analyzing data from the Water Fluoridation Reporting System (WFRS), moreover, they obtained the percentage of the US population that received optimally fluoridated drinking water (67.1%). Kairkar et al., 2021 identified three geographical areas based on different water fluoride levels, ranging from 2.5 ppm to 10 ppm.

In the studies by Riddell et al., 2019 and Barberio et al., 2017, urinary analyses were performed using an Orion PH meter with a fluoride ion selective electrode after dilution with an ionic adjustment buffer. Urinary fluoride concentration was adjusted for creatinine and specific gravity. Moreover, tap water samples were analyzed for fluoride concentrations through basic anion exchange chromatography procedure. Wang et al., 2022 collected urinary samples of the recruited children and, using a fluoride ion-selective electrode, measured the fluoride concentration and urinary creatinine. Adkins et al., 2022 examined urine samples, with ionic fluoride diffused from the specimens through the hexamethyldisiloxane (HDMS) diffusion method. Then, urinary specific gravity was calculated to correct for dilution. Bashash et al., 2018 used maternal urinary samples as a marker of prenatal fluoride exposure: in particular, the average of all available creatinine-adjusted maternal urinary fluoride (MUFcr) concentrations was calculated.

Regarding ADHD diagnosis, only 3 studies applied specifically validated questionnaires for ADHD [42,43,47]. Malin and Till 2015 collected information about the diagnosis by using the National Survey of Children’s Health (NSCH), a cross-sectional digital survey in which parents were asked questions regarding the well-being of children in their household, including whether those children had received an ADHD diagnosis. The same data were used by Perrott 2018 [45]. Instead, Adkins et al., 2022 asked parents to complete the Behavior Assessment System for Children-2 (BASC-2), a tool that consists of 16 primary measurement domains (including the Hyperactivity domain) but is not specific for ADHD diagnosis. Barberio et al., 2017 used a single item of the survey to ask whether the child was diagnosed with a learning disability and, if so, of what kind (namely Attention Deficit Disorder, no hyperactivity (ADD)/ADHD/Dyslexia/Other).

The heterogeneity of study designs has an impact on the results of the articles we included in our review: Malin and Till 2015, Riddell et al., 2019 and Khairkar et al., 2021 suggest there is a positive correlation between fluoride exposure and ADHD diagnosis. Bashash et al., 2018 draws attention to a relation between maternal fluoride exposure and inattention, whereas no association is found between maternal fluoride exposure and hyperactivity or impulse control dysfunctions. Conversely, Barberio et al., 2017 and Perrott 2018 observe no correlation between fluoride exposure and ADHD. Adkins and Wang note a positive correlation between fluoride exposure and the development of internalizing symptoms such as somatization, but they do not find any significant connection with ADHD.

## 4. Discussion

The aim of our review was to sharpen our knowledge of how fluoride can affect the risk of ADHD development in children and adolescents. The studies included here investigate the effect of early fluoride exposure on children and adolescents with different designs and methods. Chronologically, Malin and Till 2015 were the first to outline a relation between the artificial fluoridation of community water and an increase in ADHD prevalence in children and adolescents in the United States, using data from the Centers for Disease Control and Prevention (CDC) [44]. Nevertheless, several limitations emerged from this study. Its ecological design forces one to categorize fluoride exposure as exposed versus non-exposed, regardless of individual fluoride levels. Moreover, although Malin and Till 2015 adjusted their statistical model for socio-economic status, they did not adjust for several other confounders subsequently included by Perrott 2018 [45]. The latter performed multiple regression analysis on the same data as Malin and Till 2015, by including other variables considered as ADHD-related in a study by Huber et al. [50]. Interestingly, the interrelation between ADHD and the extent of fluoridation exposure was not confirmed after further adjustment for confounding factors, suggesting that a causal effect is unlikely. Nevertheless, both Malin and Till 2015 and Perrott 2018 did not exclude the presence of other environmental contaminants (such as lead or arsenic) that could interfere with their results.

Khairkar et al., 2021, conducted a case–control study in India, identifying three different areas based on fluoride levels in drinking water. They found a relation between water fluoride levels and the development of psychiatric manifestations such as ADHD, childhood disruptive mood disorder, defiant disorder and arithmetic specific scholastic skill disorder [49]. However, this study bears the limitation of not having collected data about individual fluoride levels in the enrolled subjects as well as not having excluded the presence of other environmental contaminants that could favor the development of the described symptomatology [51]. Therefore, based on the available data, we cannot rule out the possibility that risk modifying factors were not included in the statistical analysis.

Barberio et al., 2017 used data from the Canadian Health Measures Survey (Cycle 2 and 3) to investigate the relation between fluoride exposure (measured by urinary fluoride, fluoride in tap water and CWF levels) and learning disabilities. For each subsample, the Authors were able to obtain not only urinary fluoride, but also creatinine-adjusted urinary fluoride and gravity-adjusted urinary fluoride, which are thought to be more accurate due to the correction of urinary dilution [52]. However, renal function is generally not affected in the age groups under investigation [53]. When statistical analysis was performed, they found a weak but positive correlation between urinary fluoride and reported learning disabilities and neurodevelopmental disorders, including ADHD. On the other hand, the strength of such results waned with creatinine-adjusted and specific-gravity adjusted urinary fluoride values. As suggested by the Authors, the association between urinary fluoride and learning disabilities may thus not be robust [48]. In the study by Barberio et al., 2017, moreover, patients with learning disabilities were included, rather than those with an ADHD diagnosis, only. Over the past decades, there has been extensive discussion among psychiatric practitioners about the possible overlap between ADHD and specific learning disabilities (SLD) [54]. In particular, practitioners and researchers are currently discussing how to operationalize these two disorders in the light of a high impact on different settings, particularly evident within the school system [55]. Therefore, the inclusion of children with learning disorders, by assimilating them to patients with ADHD, should be considered as a potential bias.

A common limitation to most of the studies is the lack of a specific validated test for ADHD diagnosis. ADHD diagnosis is mainly clinical, made by a qualified health care provider and based on patients’ symptoms, general psychosocial situation and interpersonal as well as psychiatric history. The use of tests such as Conners’ Rating Scale can also be helpful in confirming and endorsing the diagnosis [10].

Riddell et al., 2019 used the Strengths and Difficulties Questionnaire (SDQ) to objectify ADHD diagnosis by physicians. The SDQ consists of 25 items, subdivided into five subscales: emotional problems, conduct problems, hyperactivity-inattention, peer problems, and prosocial behavior [42]. Using data from the Canadian Health Measures Survey (Cycle 2 and 3), Riddell confirmed the lack of correlation between urinary fluoride and hyperactive/inattentive symptoms, as suggested by Barberio et al., 2017 [48]. Conversely, exposure to higher levels of fluoride in tap water seemed to be associated with an increased risk of ADHD symptoms and diagnosis among Canadian adolescents. An increase of 1 mg/L in water fluoride concentration was linked with a 6.1 times higher probability of ADHD diagnosis. Although Barberio et al., 2017 and Riddell et al., 2019 had used the same survey, their results differ, probably due to the selected population: the first study focused on children aged 3 to 12 years, whereas the second one on youths aged 6 to 17 years. Riddell et al., 2019 noted that age altered the association between fluoride exposure and ADHD symptoms, suggesting that this association becomes stronger in older children. This phenomenon could be explained by the fact that the human brain is highly sensitive to environmental toxins in adolescents [28]. Alternatively, it could be related to the epigenetic impact of fluoride exposure on gene expression, which could manifest later on [56].

Wang et al., 2022 asked parents to complete the Conners’ Parent Rating Scale-Revised (CPRS-R) to support ADHD diagnosis in children aged 7–13 years. The CPRS-R uses six subscales to assess different behavioral domains: conduct problems, learning problems, psychosomatic problems, impulsive-hyperactive behaviors, anxiety, and ADHD index. Wang found no correlation between urinary fluoride and ADHD symptoms or diagnosis. However, there was a link between fluoride exposure and somatization symptoms, suggesting a neurotoxic effect of fluoride on the human brain [47]. This finding was supported by Adkins et al., 2022, who used the BASC-2 scale to determine the impact of fluoride exposure and internalizing symptoms, particularly somatization [46]. As pointed out earlier, on the other hand, Barberio et al., 2017 and Riddell et al., 2019 excluded a relation between urinary fluoride and ADHD development [42,48]. As in Riddell’s study, this outcome might change by including tap water fluoride samples as an alternative measure of exposure. This stronger relation may be linked to the smaller variation of fluoride concentrations in water supplies compared to urinary fluoride levels, especially urinary spot samples that are more prone to fluctuating due to the rapid clearance of fluoride. In particular, urinary fluoride assessment may detect acute fluoride exposures, especially after professional exposure, consumption of fluoride-rich drinks or swallowing toothpaste prior to urine sampling.

Unlike the studies described above, Bashash et al., 2018. used data from the Early Life Exposures to Environmental Toxicants (ELEMENT) cohort to verify an association between prenatal fluoride exposure and the development of ADHD. Maternal urinary fluoride samples were used as biomarkers of prenatal fluoride exposure. For ADHD assessment, CPRS-R was completed by mothers, while the Conners’ Continuous Performance Test (CPT-II) was administered to enrolled children. Moreover, the DSM-IV Inattention Index, DSM-IV Hyperactive-Impulsive Index, and DSM-IV Total Index (inattentive and hyperactive-impulsive behaviors combined) were used to confirm the diagnosis. Bashash et al., 2018. noted that higher prenatal fluoride exposure, as measured by MUFcr, corresponded to more ADHD-like symptoms on the CRS-R, particularly related to inattention: a 0.5 mg/L-higher level of fluoride in maternal urine resulted in higher scores on the CRS-R for DSM-IV Inattention and Cognitive Problems and Inattention. On the other hand, maternal urinary fluoride was not associated with hyperactivity and impulse control [43].

Differences in study designs, fluoride exposure measures and methods for diagnosing ADHD may account for the heterogeneity in our results. However, evidence of neurotoxic effects on brain development has been repeatedly supported, particularly in terms of IQ performance [31,32,57,58]. Finally, the low number of studies investigating the association between fluoride exposure and ADHD is a limitation of our review.

## 5. Conclusions

Current epidemiological evidence indicates that fluoride exposure may have neurotoxic effects on neurodevelopment, including behavioral alterations, cognitive impairment and psychosomatic issues. However, the heterogeneity in study designs and results from human studies did not allow us to reliably identify fluoride exposure as a risk factor for ADHD development. More rigorous studies are needed to provide conclusive evidence of an etiologic association between pre- or post-natal fluoride exposure and ADHD.

## Figures and Tables

**Table 1:** Search strategies in online databases.

Database	Search Strategy
PubMed	((fluoride) AND ((ADHD) OR (attention deficit hyperactivity disorder))
Embase	(‘fluoride’/exp OR fluoride) AND (‘adhd’/exp OR adhd OR ‘attention deficit hyperactivity disorder’/exp OR ‘attention deficit hyperactivity disorder’ OR ((‘attention’/exp OR attention) AND deficit AND (‘hyperactivity’/exp OR hyperactivity) AND (‘disorder’/exp OR disorder)))
Web of Science	fluoride AND ADHD

**Table 2:** Characteristics of the studies included in the review.

Reference	Country	Study Type	Aim of the Study	Population	Number of Samples	Fluoride Exposure Method	Average Fluoride Levels	ADHD Assessment	Main Findings	Adjustment Factors
Bashash et al., 2018 [43]	Mexico	Cohort study (ELEMENT project)	To assess the relation between prenatal exposure to fluoride and ADHD	Mother-child pairs	210	Maternal Urinary fluoride adjusted for creatinine (MUFcr)	The overall mean level of MUFcr averaged across all trimesters was 0.85 mg/L, with an Interquartile Range (IQR) of 0.46 mg/L	CRS-R or CPT-II or DSM-IV Hyperactivity-Impulsivity scale, DSM-IV ADHD scale, DSM-IV Inattention scale	Association between MUFcr and CRS-R for DSM-IV Inattention and Cognitive problems and inattention, not hyperactivity or impulse control	Smoking, maternal education, child sex, HOME score, exposure to other contaminants
Malin and Till 2015 [44]; Perrott 2018 [45]	USA	Cross-sectional ecological survey [National Survey of Children’s Health (2003, 2007, 2011)]	To examine the relation between exposure to fluoridated water and ADHD prevalence among children and adolescents	Children aged 4–17	2003: 79,264 2007: 73,123 2011: 76,015	Centers for Disease Control and Prevention (CDC) website provide data on the number of people receiving fluoridated water from public water supplies in each of America’s 50 states	-	Parent reported health care provider-diagnosed ADHD	Prevalence of ADHD increased from 7.8% in 2003 to 11% in 2011. Water fluoridation prevalence was positively associated with ADHD. The association between water fluoride exposure and ADHD incidence disappears once elevation is included in the multiple regression analysis.	Socioeconomic status in the first report and mean state elevation, median household income, smoking prevalence 2013, water fluoridation in 1992, obese youth average 2003–2004, longitude, home ownership, per capita personal income 2009, low birth rate 2005, bachelor’s degree 2000, age over 65 in the fully adjusted model
Riddell et al., 2019 [42]	Canada	Cross-sectional survey [Canadian Health Measures Survey (Cycles 2 and 3)]	To examine the relation between urinary and tap water fluoride concentration and attention-related outcomes	Children aged 6–17	1877 UF 1722 Community Water Fluoridation (CWF) status 980 tap water sample	Urinary fluoridation (UF), city fluoridation status, tap water fluoride	The mean urinary fluoride adjusted for specific gravity (UFSG) concentration was 0.61 mg/L among the 1877 youths from Cycles 2 and 3	ADHD diagnosis SDQ Hyperactivity-inattention subscale	Children exposed to higher tap water fluoride levels had a higher risk of receiving an ADHD diagnosis. UF levels were not associated with ADHD or symptom-related	Sex, age, ethnicity, BMI, level of parental education, household income, cigarette smoke exposure, blood lead level
Adkins et al., 2022 [46]	USA	Cross-sectional study [CCAAPS]	To examine the association between UF and internalizing symptoms among adolescents, also evaluating sex differences	Adolescents	334	UF	UF concentrations at Age-12 visit was 0.88 (0.36) mg/L	Parent reported BASC-2 scale	UF concentration are positively associated with internalizing behaviors, particularly somatization.	Race, sex, age, total family income at age-12 visit, maternal depression, serum cotinine, PRQ relational frustration
Wang et al., 2022 [47]	China	Cross-sectional study	To investigate the relation between fluoride exposure and behavioral outcomes among children	Primary schools’ children	325	UF	The mean ± SD levels of UF was 1.54 ± 0.89 mg/L	CPRS-48 (ADHD Index)	UF associated with psychosomatic problems, no correlation with ADHD	Age, BMI, urinary creatinine, sex, parents migrated
Barberio et al., 2017 [48]	Canada	Cross-sectional survey [Canadian Health Measures Survey]	To examine the association between fluoride exposure and learning disabilities.	Children aged 3–12	Cycle 2 = 1120 Cycle 3 = 1101	UF, creatinine-adjusted urinary fluoride, gravity-adjusted urinary fluoride, tap water sample	UFSG Cycle 2 fluoride subsample: 37.78 µmol/L Cycle 2 constrained fluoride subsample = 43.46 µmol/L Cycle 3 fluoride subsample: 34.25 µmol/L Cycle 3 constrained fluoride subsample: 40.71 µmol/L	Response to items of the survey	No association between UF and learning disabilities and ADHD diagnosis.	Sex, age, household education, household income adequacy
Khairkar et al., 2021 [49]	India	Case–control study (Letter to editor)	To analyze the outcome of spectral severity of water fluoride levels in adolescents on their neurocognitive and neuropsychiatric disorders	Adolescents aged 11–15	150	Level of fluoride in community water	-	Not specified	Significant association between fluoride exposure and ADHD, disruptive behaviors, defiant disorder and scholastic arithmetic skill disorders	Not specified

Abbreviations: ADHD: Attention deficit hyperactivity disorder. BMI: body mass index. ELEMENT: Early Life Exposure in Mexico to Environmental Toxicants project. UF: Urinary fluoridation. UFSG: urinary fluoride adjusted for specific gravity. MUF: Maternal Urinary fluoride. MUFcr: Maternal Urinary fluoride adjusted for creatinine. CDC: Centers for Disease Control and Prevention. CRS-R: Conners’ Rating Scales—Revised. CPT-II: Conners’ Continuous Performance Test. DSM-IV: Diagnostic and Statistical Manual of Mental Disorders—4th Edition. CWF: Community Water Fluoridation. CCAAPS: Cincinnati Childhood Allergy and Air pollution study. BASC-2: Behavior Assessment System for Children-2. CPRS-48: Conner’s Parent Reporting Scale-Revised 48.

## Data Availability

The data presented in this study are available in the article.
